# A Coaxial Cable Fabry-Perot Interferometer for Sensing Applications

**DOI:** 10.3390/s131115252

**Published:** 2013-11-07

**Authors:** Jie Huang, Tao Wang, Lei Hua, Jun Fan, Hai Xiao, Ming Luo

**Affiliations:** 1 Department of Electrical and Computer Engineering, Center for Optical Materials Science and Engineering Technologies, Clemson University, Clemson, SC 29634, USA; E-Mail: jieh@clemson.edu; 2 Department of Electrical and Computer Engineering, Missouri University of Science and Technology, Rolla, MO 65409, USA; E-Mails: twkmf@mst.edu (T.W.); lhgn8@mst.edu (L.H.); jfan@mst.edu (J.F.); 3 Habsonic LLC, 1105 Hauck Drive, Rolla, MO 65401, USA; E-Mail: ming.luo@habsonic.com

**Keywords:** Fabry-Perot interferometer, coaxial cable sensor, structural health monitoring, temperature and strain measurement

## Abstract

This paper reports a novel coaxial cable Fabry-Perot interferometer for sensing applications. The sensor is fabricated by drilling two holes half-way into a coaxial cable. The device physics was described. The temperature and strain responses of the sensor were tested. The measurement error was calculated and analyzed.

## 1. Introduction

In the past decades, fiber optic sensors have found many successful applications due to their unique advantages such as low loss, light weight, compactness, high resolution, immunity to electromagnetic interference, remote operation, and multiplexing capability [1,2]. For example, fiber Bragg gratings (FBG) have been widely investigated and successfully applied in sensing applications since their first demonstration in 1978 [3]. FBGs have shown many advantages in sensing applications, such as high resolution inherent self-referencing capability, and easy multiplexing. Unfortunately, optical fibers are fragile and fiber optic sensors have a relative small dynamic range due to the limited deformability of silica glass. Even with rigorous packaging, fiber sensors can easily break when they are subject to large strains (about 0.4mε or 0.4%) and/or a shear force, causing serious challenges for sensor installation and operation [4]. As such, the applications of fiber optic sensors are limited in the heavy duty or large strain (e.g., more than 2%) measurements which are commonly desired in structural health monitoring (SHM).

One solution to the problem is to find and use another type of optical cable, for example a polymer optical fiber (POF), as the transmission medium with improved flexibility and robustness to survive the large strains. However, POFs have a large signal loss in optical communication frequencies and a large core size which supports many modes [5–7]. As a result, it is difficult to obtain a POF sensor with high signal quality. Although great efforts have been made to improve the performance of POF [8,9], the commercially available single-mode POFs currently are still expensive. Micro-structured POFs are even more difficult to fabricate [10–12].

From the electromagnetic point of view, a coaxial cable performs a similar function as an optical fiber by transmitting an electromagnetic signal over a long distance. A typical coaxial cable consists of an inner and outer conductor sandwiched by a tubular insulating layer with a high dielectric constant. Governed by the same electromagnetic (EM) theory, a coaxial cable and an optical fiber share the common fundamental physics. However, the EM wave frequencies supported by them are quite different. The optical frequency is orders of magnitude higher than the radio frequency (RF). Over the years, optical fiber and coaxial cable technologies have evolved along quite different paths, resulting in unique devices of their own right. In comparison with an optical fiber, a coaxial cable can survive a large strain and is relatively insensitive to lateral force or bending.

Inspired by the well-known FBG, we have recently successfully developed a new coaxial cable Bragg grating (CCBG) sensor [13]. The large dynamic range, robustness and high resolution of the CCBG sensor provide a very promising and effective solution for SHM [14]. However, the CCBG has a long gauge length (∼ 1 m), and as a result, the spatial resolution of the CCBG sensor is limited.

**Figure 1.**
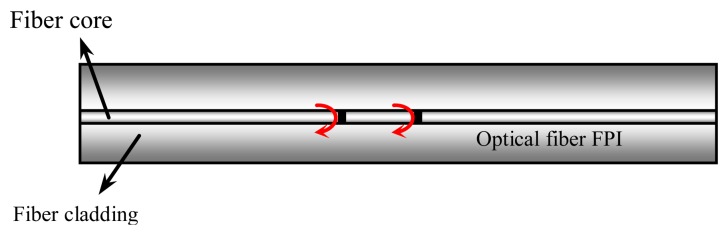
Schematic of an optical fiber based FPI.

In this paper, we propose a new coaxial cable sensor platform to achieve high spatial resolution. The new sensor platform is inspired by the optical fiber inline Fabry-Perot interferometer (FPI) [15,16]. FPIs typically have comparable sensitivity to FBGs, but a much shorter length than FBGs. As shown in Figure 1, a FPI consists of a cavity formed by two reflectors with a typical separation of tens to hundreds of micrometers. Light waves reflected at the two reflectors have a different time delay, resulting in an interference signal (e.g., an interferogram in spectrum domain) that can be demodulated to find the optical length of the cavity. The variations in ambient temperature and/or strain will change the physical length or material properties of the medium between the two reflectors, leading to a shift in the interference pattern. This shift can be measured to find the ambient temperature or strain change.

Similarly, we can engineer partial reflectors inside a coaxial cable to construct a coaxial cable Fabry-Perot interferometer (CCFPI). Here, we report the design and fabrication of such a CCFPI and describe its application for temperature and strain sensing, which are the two most important aspects in SHM.

## 2. Principle of CCFPI

### 2.1. Fundamental Physics

**Figure 2.**
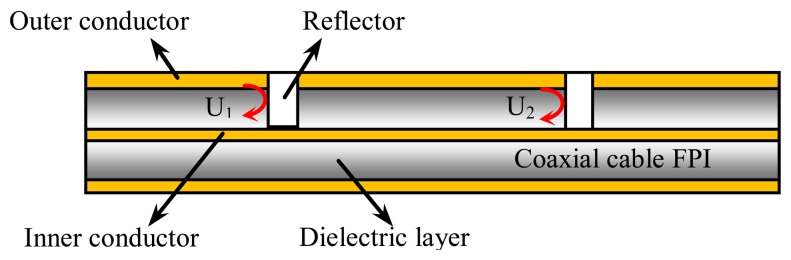
Schematic of a CCFPI.

As shown in Figure 2, a CCFPI consists of a pair of partial reflectors separated by millimeters to centimeters. The EM wave traveling inside the cable is partially reflected at the first reflector while the remaining energy transmits through to reach the second reflector. At the second reflector, the EM wave is again partially reflected. The two reflected waves travel backwards and interfere coherently to generate an interference signal. When observed in the spectrum domain, the interference signal manifests itself as an interferogram.

The two reflectors can be engineered to have a low reflectivity. As a result, multiple reflections between two reflectors can be negligible in the calculation. Assuming the amplitude reflection coefficients of the two reflectors are the same, the two reflected waves (*U_1_* and *U_2_*) can be written as:

(1) $$U_{1}=\Gamma (f)e^{-az}\cos(2\pi \;ft),U_{2}=\Gamma (f)e^{-az}\cos[2\pi \;f(t+\tau )],\tau =\frac{2d\sqrt{\varepsilon_{r}}}{c}$$

where *Γ(f)* is the amplitude reflection coefficient of the reflector; *f* is frequency of the EM wave traveling inside the cable; *α* is the propagation loss coefficient; *z* denotes the cable axial direction; *τ* is time delay between the two reflected waves; *d* is the distance between two reflectors; *ε_r_* is the relative permittivity of the inner dielectric material of the cable; *c* is the speed of light in vacuum.

The two reflected waves have a time delay and the delay is associated with the distance between the two reflectors and the phase velocity of the wave. The interference signal (*U*) is the summation of the two reflected waves, which can be written as:

(2) $$U=2\cdot \Gamma (f)e^{-az}\cos(2\;\pi \;f\;\tau )\cos[2\;\pi \;f(t+\tau )]$$

Equation (2) describes a wave with its amplitude given by $2\cdot \Gamma (f)e^{-az}\cos(2\pi \;f\;\tau )$ and its phase of 2*πfτ*. The amplitude and phase vary as functions of frequency and the delay. In essence, the amplitude of spectrum varies sinusoidally as the frequency of wave is scanned.

**Figure 3.**
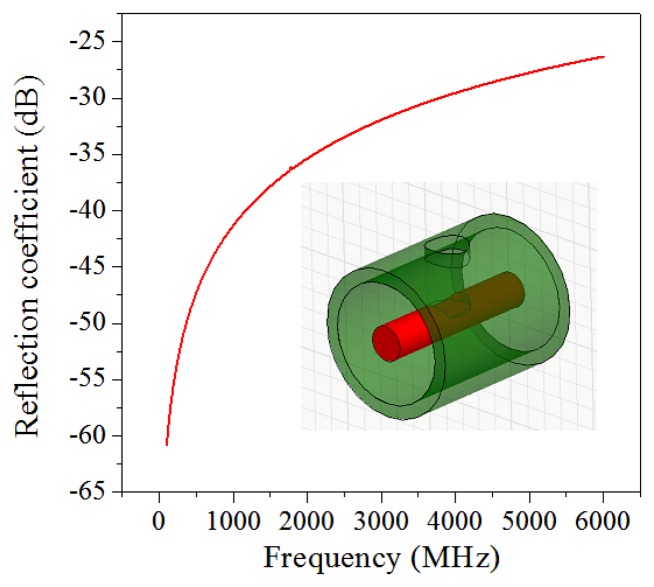
Calculated reflection coefficient in magnitude of the proposed reflector on a coaxial cable.

In Equation (2), the only unknown parameter is the reflection coefficient *Γ(f)*. A partial reflector can be generated by introducing an impedance discontinuity in a coaxial cable. There are many methods to implant the impedance discontinuity. In our preliminary research, we used a simple method by drilling a cylindrical air hole into a coaxial cable without touching the inner conductor to avoid significant signal loss. The depth and size of the air hole can be varied to change the reflectivity. The reflection coefficient can be numerically simulated by a commercial full-wave solver including magnitude and phase at discrete frequency. Figure 3 plots that the reflection coefficient (in magnitude) increased as the interrogated frequency increased.

**Figure 4.**
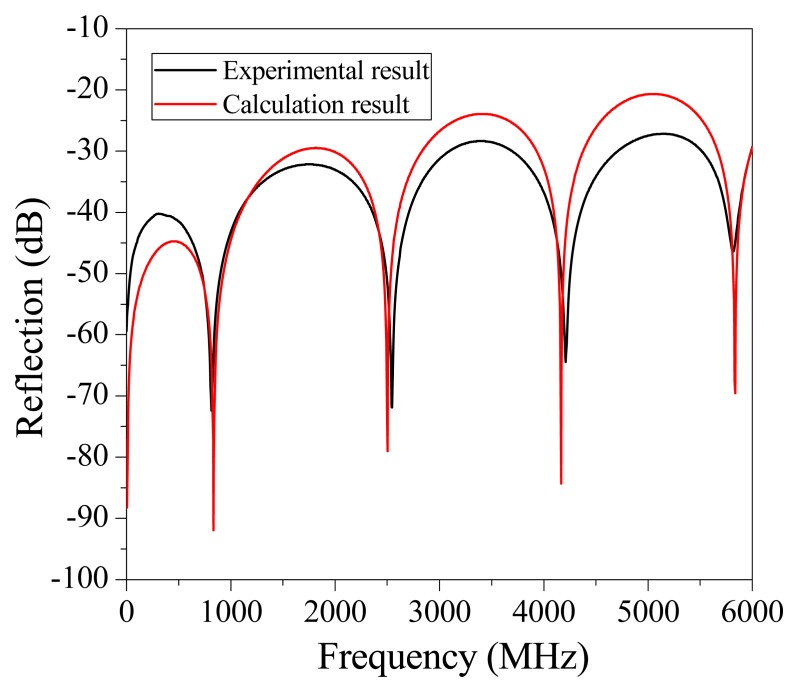
Measured and simulated interferogram of a CCFPI.

To numerically calculate the interferogram of a CCFPI, the relative permittivity of the dielectric material was set to be 2.25 and α was 0.04 dB/m in the bandwidth of 0 to 6 GHz. The distance between two reflectors (*d*) was 60 mm. By substituting the calculated reflection coefficient in Figure 3, the interferogram of a typical CCFPI was plotted in Figure 4 (red curve). Several resonant dips can be observed including fundamental frequency and its harmonics. The amplitude of the constructive interferences from 0 to 6 GHz followed the trends shown in Figure 2. The signal-to-noise ratio (SNR) was over 40 dB, indicating that it can be used for sensing after proper calibration. The quality factor (Q-factor) was about 5. Typically, the Q-factor for a FPI is associated with the reflectivity of each reflector and the transmission loss inside the cavity. The Q-factor increases as reflectivity increases or the loss decreases. People can make the Q-factor up to several thousand due to higher reflectivity and low loss. However, the reflection coefficient of each reflector in a CCFPI needs to be designed as low as possible (∼−30 dB in Figure 2) due to the purpose of multiplexing capability.

The above investigation into device physics reveals that the resonant behavior mainly results from the coherent interference. The reflections are generated by impedance discontinuities as a result of interruption in material properties such as the permittivity and permeability or in cable parameters such as the resistance, capacitance or inductance. As such, there are many potential methods to create impedance discontinuity in a coaxial cable besides hole-drilling method. In addition, the reflection coefficient of discontinuity can be designed to obtain more complex reflection profile and this would require more involved analysis.

### 2.2. Measurement Error Analysis

In practical measurement, measurement errors often occur due to the lack of data information or noisy measurement, and are often difficult to detect since the true value of the parameter under test is unknown. However, it can be numerically analyzed through proper assumption.

According to Equation (2), the environmental parameters change (e.g., strain or temperature) will cause a change in time delay. Subsequently, the interference pattern will change. By tracking the shift in the interferogram, the changes of parameters can be found. In real operation, it is easy to follow the shift in the resonant frequency. From Equation (2) the N*th* resonant frequency can be deduced, which is:

(4) $$f_{N}=\frac{N}{\tau }=\frac{Nc}{2d\sqrt{\varepsilon_{r}}}$$

In general, the stretch of the cable will cause an elongation to the cable and a decreasing to the dielectric constant due to the photoelasticity effect. These are the dominant factors that will further influence the interferogram. As a result, the applied strain (*ε*) can be expressed in terms of changes in distance and relative permittivity of the material (*Δε_r_*) using the following equation:

(5) $$\varepsilon =\frac{\Delta d}{d}\;\text{and}\;\frac{\Delta \in_{r}}{\in_{r}}≃-P_{\text{eff}}\varepsilon$$

where *P_eff_* represents the effective Pockels coefficient of the inner dielectric material of the coaxial cable. The N*th* resonant frequency shift (*Δf_N_*) can be deduced from Equations (3) and (4) as follows:

(6) $$\Delta f_{N}=\frac{\partial f_{N}}{\partial d}\Delta d+\frac{\partial f_{N}}{\partial \in_{r}}\Delta \in_{r}$$

The applied strain (ε) in terms of the N*th* resonant frequency change (*Δf_N_*) can be further derived from Equation (5):

(7) $$\varepsilon =\frac{2\Delta f_{N}}{f_{N}(\in_{r}P_{\text{eff}}-2)}$$

Equation (6) describes that the applied strain can be calculated by simply measuring the N*th* resonant frequency shift (*Δf_N_*) while assuming that the other parameters in the equation are initially known. The measurement error of strain (*Δε_error_*) can be deduced from Equation (6) as follows:

(8) $$\Delta \varepsilon_{\text{error}}=\left|\frac{\partial \varepsilon }{\partial \Delta f_{N}}\Delta f_{N\_\text{error}}\right|=\left|\frac{2}{f_{N}(\in_{r}P_{\text{eff}}-2)}\Delta f_{N\_\text{error}}\right|$$

where *Δf_N_error_* represents the measurement error of the frequency shift. This value can be calibrated using high accuracy instrument. The measurement range (observation bandwidth of the spectrum) can be initially set to be small so that the *Δf_N_error_* can be small if the sampling point of the instrument is fixed. Another observation is that the higher interrogated frequency (*f_N_*) will have lower measurement error.

When the CCFPI is subjected to temperature variation, both the relative permittivity of the material and the length (*d*) will change due to the effect of temperature on the dielectric constant and the thermal expansion of the material, respectively. By using the same derivation method for strain measurement error, the temperature measurement error (*ΔT_error_*) can be expressed as follows:

(9) $$\Delta T_{\text{error}}=\left|\frac{2}{f_{N}(2\alpha_{\text{CTE}}+\sqrt{\in_{r}}\alpha_{TD})}\Delta f_{N\_\text{error}}\right|$$

where *α_CTE_* and *α_TD_* are the coefficients of thermal expansion and thermal effect on dielectric constant, respectively.

## 3. Experimental Results

### 3.1. Hole-Drill Method

In order to precisely control the shape and depth of the drilled hole, a computer numerical controlled (CNC) drilling operator (Model 2000, P/N 8020A, Sherline, Carlsbad, CA, USA) was used, where the minima feeding step of the three axes (x, y, z) is 10 μm. A vector network analyzer (VNA HP 8753ES, Santa Clara, CA, USA) was used to monitor *in situ* the reflection spectrum during fabrication process. One end of the coaxial cable (50 Ω, RG-58, Jamco Electronics, Belmont, CA, USA) was launched to one port of VNA and the other end was matched with a 50 Ω terminator. A drilling bit with diameter of 1/12 inch was used. All the machines including VNA were controlled by a computer. The distance between two holes was 60 mm. The drilling depth was 2.1 mm and the out diameter of the cable was 5 mm. The coaxial cable was properly calibrated by VNA before fabrication. The VNA was configured to with an observation bandwidth from 100 kHz to 6 GHz, a total of 1,601 sampling points and intermediate frequency bandwidth (IFBW) of 10 kHz. A band-pass gating was applied in time domain (after Fourier transfer to the microwave spectrum, a built-in function in VNA) to select the two reflections of drilled holes and suppress multiple reflections from two ends of the coaxial cable.

Figure 4 plots the measured interferogram (black curve) of a CCFPI within the frequency range of 100 kHz to 6 *versus* the calculated interferogram (red curve). Several resonant frequencies can be observed including fundamental and harmonics. The SNR is over 40 dB. The Q-factor is about 5. The SNR and Q-factor match exactly well with the calculated data, indicating that the hole-drilling method did not incur in any extra loss to the cable. The resonant frequencies cannot be exactly matched because the relative permittivity of the inner dielectric material is frequency dependent, which is the similar with the chromatic dispersion in optics.

### 3.2. Temperature and Strain Measurement

To demonstrate the capability of using CCFPI as a sensing device in SHM, temperature and strain measurements were conducted. The CCFPI used for temperature measurement had a distance of 60 mm. The VNA was configured to acquire the resonant frequency of ∼4.2 GHz with an observation bandwidth from 3.6 to 4.8 GHz. The CCFPI was placed in a tubular furnace. The temperature was raised from 30 °C to 90 °C with an increasing step of 10 °C. The rising time for each step was set to be 5 °C/min. For each temperature point, the reflection spectrum was measured multiple times consecutively, and the averaged spectrum was applied to find the center frequency of the resonant peak. Fourth-order polynomial curve-fitting was applied to smooth the resonant peak for further improvement of the measurement accuracy.

**Figure 5.**
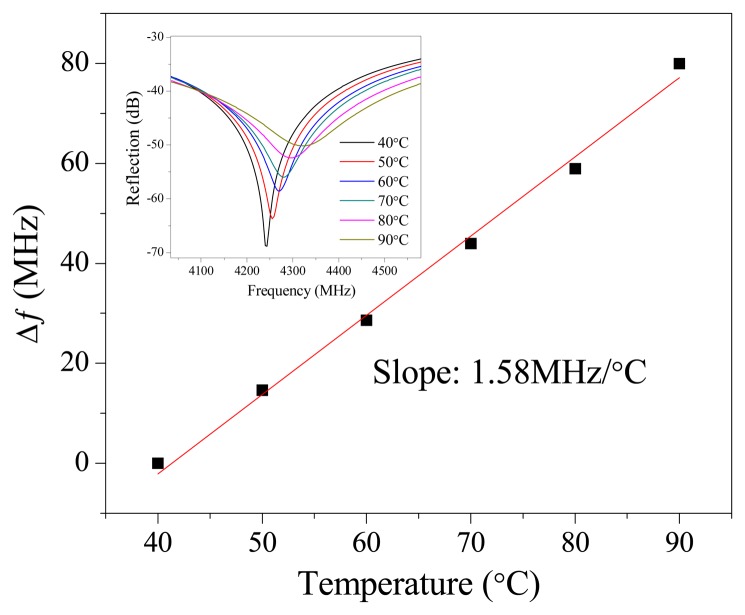
Resonant frequency shift as a function of ambient temperature. Inset: Shift in reflection spectra as the ambient temperature increases.

Figure 5 plots the change in resonant frequency as a function of the ambient temperature and the inset in Figure 5 plots the shift in reflection spectra as the ambient temperature increases. The spectra shift to higher frequency region indicating that the effective length decreases as the temperature increases. The effective length is associated with the physical length between two reflectors and the relative permittivity of the inner dielectric layer. The decreasing in effective length indicates that the relative permittivity decreases as temperature increases. As a result, the change in relative permittivity is the dominant factor when CCFPI is subjected to temperature variation. The Q-factor of the dips decreased as the temperature increased indicating that the propagation loss between two reflectors increased. The resonant frequency increases almost linear with a slope of 1.58 MHz/°C as the temperature increased. The linear temperature-frequency shift relation indicates that CCFPI can be used as a sensor for temperature after it is properly calibrated.

**Figure 6.**
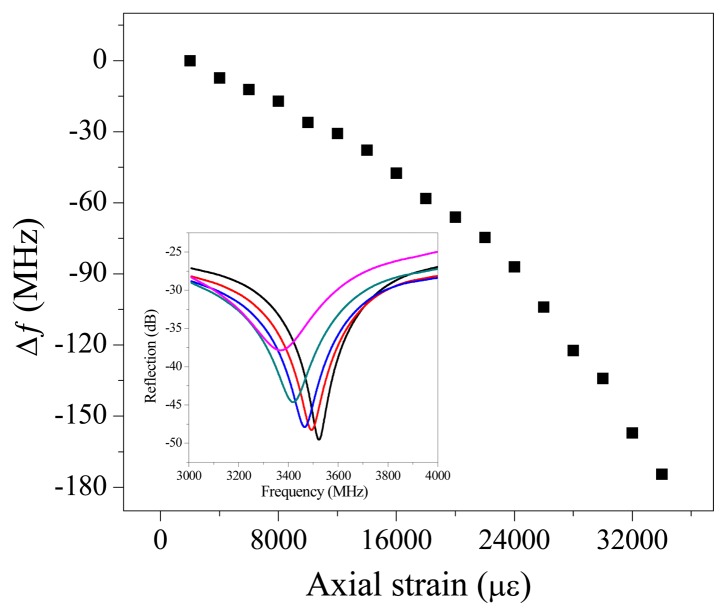
Resonant frequency shift as a function of strain. Inset: Shift in reflection spectra as strain increases.

It also has the potential in large strain measurement because the stretch of the CCFPI will directly increase the distance between two reflectors, resulting in a linearly decrease of the resonant frequency. In the strain test, the CCFPI had a distance of 70 mm and was fixed onto two translation stages. The gauge length (distance between two stages) was 500 mm. A pre-strain was initially applied to the cable before testing. After elongating the gauge length at a step of 1 mm, corresponding to a strain increase of 2,000 με (0.2%), the reflection spectrum was acquired through VNA. Eighteen increasing steps or a total strain of about (34,000 με) 3.4% were applied to the cable. Figure 6 plots the change in resonant frequency as a function of the applied strain. The inset plots the shift in reflection spectra as the axial strain increased. The spectra shifted to the lower frequency range, which can be predicted in Equation (2). The quasi-linear strain-frequency shift relation (∼3.3 kHz/με) indicates that CCFPI can be used as a sensor for large strain measurement after it is properly calibrated.

## 4. Conclusions

To summarize, this paper reports a coaxial cable Fabry-Perot interferometer fabricated by drilling two holes half-way into the cable. The open hole resulted in an impedance discontinuity and partial reflection of EM wave propagating inside the cable. The two holes/reflectors produced interference patterns with multiple resonant frequencies. To understand the device physics, the reflection coefficient of the open hole was calculated using finite element analysis and the device was modeled based on traditional EM theory. The theoretic calculation matched well with the experimental result. The measurement error was also investigated. Temperature and strain responses of CCFPI were experimentally demonstrated. Although in this paper the hole-drilling method was used to demonstrate the CCFPI concept and temperature sensing was selected as an example application, there exist many other methods to fabricate CCFPI. It is predicable that the CCFPI has advantages of robustness, high spatial resolution and large strain capability. These unique features may enable potential applications in SHM.
